# The Position of the Dentist

**Published:** 1880-10

**Authors:** 


### ARTICLE VII.

### *The Position of the Dentist.*

Considerable feeling has been excited in England over the views which the *Lancet* has expressed in regard to the professional position of the dentist. That conservative journal has taken the ground that the dentists, or those who hold, as the bulk of them do, a diploma in dental surgery, are not to be considered in any sense members of the medical profession. Furthermore, it is not permitted them to contribute to the columns of the *Lancet*, because they are professionally inferior. This last prohibition was brought about through an invitation which the editor issued to his readers to furnish information upon the subject of the extraction of teeth and putting them back in the jaw. None but medical men, or dentists who had a medical degree or license, were allowed to contribute to the discussion. It appears that the dentists of England have, of late years, become a body of considerable scientific attainments and prominence. A good many write themselves "surgeons practising dentistry," and are rather above the oldfashioned dissyllable which their fathers used. There has been much effort made to show that dentistry is no longer a mechanical trade, but a branch of medical science, which deserves to rank with other specialties, like ophthalmology or dermatology. Although these efforts to elevate the art deserve every encouragement, it will be acknowledged, we think, by most, that the *Lancet* is right in refusing, as yet, to recognize dentistry as a medical specialty. No class of persons has any right to claim membership in the medical profession, or to presume to form any branch of it, until such persons are doctors of medicine themselves. It is in this respect that dentists differ from oculists and gynecologists, etc. The latter must first get their medical diplomas. If dentists would also first get such a degree, they could justly claim every equality with other members of the profession; and such equality is, indeed, already granted everywhere. It is a fact, however, that the demand for highly educated dentists is not yet great enough to justify more than a small proportion of that profession from getting a medical degree. The degree of dental surgeon which so many do get in this country insures a good educational training and a fair amount of medical knowledge. It is making the dental profession one which is much more respected, and which is doing excellent scientific work. This diploma in dental surgery, however, is not enough to entitle him to the claim of practising a surgical specialty.

The attempt, in England, to elevate the standard of dentist by law has led to a rather curious result, if we may believe the statements of Dr. Archibald H. Jacobs, of Dublin, in the *Medical Press*. He asserts that by this law, recently enacted, 4,806 persons became admitted to the profession of dentistry, of whom only 531 possessed any recognized medical, surgical, or dental qualification. The law gives them the privilege of calling themselves surgeon-dentists, and puts them, officially and legally, on a par with surgeons. Considerable dissatisfaction has naturally been felt.—*Medical Record.*

---

### EDITORIAL, ETC.

